# Hexokinase-linked glycolytic overload and unscheduled glycolysis in hyperglycemia-induced pathogenesis of insulin resistance, beta-cell glucotoxicity, and diabetic vascular complications

**DOI:** 10.3389/fendo.2023.1268308

**Published:** 2024-01-16

**Authors:** Naila Rabbani, Paul J. Thornalley

**Affiliations:** ^1^ College of Medicine, Qatar University, Doha, Qatar; ^2^ College of Health and Life Sciences, Hamad Bin Khalifa University, Doha, Qatar

**Keywords:** hyperglycermia, glucose metabolism, insulin resistance, glucotoxicity, diabetes, vascular complications

## Abstract

Hyperglycemia is a risk factor for the development of insulin resistance, beta-cell glucotoxicity, and vascular complications of diabetes. We propose the hypothesis, hexokinase-linked glycolytic overload and unscheduled glycolysis, in explanation. Hexokinases (HKs) catalyze the first step of glucose metabolism. Increased flux of glucose metabolism through glycolysis gated by HKs, when occurring without concomitant increased activity of glycolytic enzymes—unscheduled glycolysis—produces increased levels of glycolytic intermediates with overspill into effector pathways of cell dysfunction and pathogenesis. HK1 is saturated with glucose in euglycemia and, where it is the major HK, provides for basal glycolytic flux without glycolytic overload. HK2 has similar saturation characteristics, except that, in persistent hyperglycemia, it is stabilized to proteolysis by high intracellular glucose concentration, increasing HK activity and initiating glycolytic overload and unscheduled glycolysis. This drives the development of vascular complications of diabetes. Similar HK2-linked unscheduled glycolysis in skeletal muscle and adipose tissue in impaired fasting glucose drives the development of peripheral insulin resistance. Glucokinase (GCK or HK4)-linked glycolytic overload and unscheduled glycolysis occurs in persistent hyperglycemia in hepatocytes and beta-cells, contributing to hepatic insulin resistance and beta-cell glucotoxicity, leading to the development of type 2 diabetes. Downstream effector pathways of HK-linked unscheduled glycolysis are mitochondrial dysfunction and increased reactive oxygen species (ROS) formation; activation of hexosamine, protein kinase c, and dicarbonyl stress pathways; and increased Mlx/Mondo A signaling. Mitochondrial dysfunction and increased ROS was proposed as the initiator of metabolic dysfunction in hyperglycemia, but it is rather one of the multiple downstream effector pathways. Correction of HK2 dysregulation is proposed as a novel therapeutic target. Pharmacotherapy addressing it corrected insulin resistance in overweight and obese subjects in clinical trial. Overall, the damaging effects of hyperglycemia are a consequence of HK-gated increased flux of glucose metabolism without increased glycolytic enzyme activities to accommodate it.

## Introduction

Insulin resistance, diabetes mellitus, and vascular complications of diabetes have become a major cause of morbidity and mortality in westernized societies. In 2021, there were 529 million people living with diabetes worldwide, of which 96% were patients with type 2 diabetes mellitus (T2DM) and the remaining patients with type 1 diabetes mellitus (T1DM). This is projected to increase to over 1.3 billion by 2050 (1). Prediabetes, defined by increased plasma glucose levels intermediate between those of euglycemia and diabetes, develops earlier and predisposes to the development of T2DM (2). In prediabetes, plasma glucose may be increased in fasting and postprandial phases—impaired fasting glucose (IFG) and impaired glucose tolerance (IGT), respectively (2). In 2021, the global prevalence of IFG was 298 million and IGT 464 million and are projected to increase to 414 million and 638 million by 2045, respectively (3). Insulin resistance is the primary initiating metabolic defect driving the development of prediabetes and T2DM (4, 5). It is defined as “a reduced response of target tissues to insulin, compared with subjects with normal glucose tolerance” (6). The main target tissues of insulin are skeletal muscle, liver, and adipocytes. Insulin resistance has high prevalence in Westernized countries: in the USA, the estimated prevalence of insulin resistance in the adult population is approximately 50% (7). Insulin resistance is often present many years before diabetes develops (8, 9), increasing dysglycemic stress in pancreatic beta-cells, hyperinsulinemia, and accelerating decline in beta-cell mass and function leading to the development of T2DM (6, 10).

With high prevalence of diabetes, there is a related high prevalence of vascular complications of diabetes—diabetic kidney disease (DKD), diabetic retinopathy, diabetic neuropathy, and increase risk of cardiovascular disease (CVD). Vascular complications of diabetes affect 30%–50% of patients of diabetes, producing increased morbidity, decreased quality of life, and premature mortality, the latter mainly through increased fatal CVD (11–14). There are also similar vascular complications in prediabetes of lower prevalence, as judged by their presence at diagnosis of T2DM (15).

Hyperglycemia, particularly increased fasting plasma glucose (FPG), is a risk factor for progression of insulin resistance and decline in beta-cell function leading to the development of T2DM (16, 17). It is also a risk factor for the development of vascular complications of diabetes (18, 19). Hyperglycemia-induced metabolic dysfunction of glucose metabolism underlies this relationship. There is currently no pharmacotherapy addressing this—most current drug treatment addressing rather hyperglycemia itself by hypoglycemic agents and incretin mimetics (20). Pharmacotherapy of DKD is mainly based on drugs directed to the renin–angiotensin–aldosterone system, severe diabetic retinopathy is treated by panretinal laser photocoagulation and anti-vascular endothelial growth factor therapy to decrease risk of visual loss, and diabetic neuropathy treatment involves mainly analgesics for pain relief. The risk of microvascular and macrovascular complications is addressed more generally by controlling hyperglycemia, blood pressure, and lipids (21–23), but there remains substantial risk of vascular complications development and progression to severe symptoms.

Herein, we re-examine experimental evidence of the origin of hyperglycemia-induced metabolic dysfunction, propose a new hypothesis in explanation of the initiation of metabolic dysfunction in hyperglycemia—hexokinase-linked glycolytic overload and unscheduled glycolysis—explaining its advantages of the over existing explanations and the promise for improved understanding of pathogenesis and therapeutics development.

## Hexokinases—catalyzing the first committed step of glucose metabolism

Hexokinases (HKs) catalyze the first step of glucose metabolism, the conversion of glucose to glucose-6-phosphate (G6P) (24): glucose + MgATP → G6P + MgADP. They are gatekeepers at the entry of glucose into metabolism. Thereafter, G6P is the substrate for onward metabolism to pyruvate through glycolysis, to ribose-5-phosphate in the pentosephosphate pathway (PPP), and to glucose-1-phosphate for glycogen synthesis. G6P is reconverted to glucose by G6P phosphatase (G6Pase) in gluconeogenesis. G6P is mainly utilized in glycolysis in all tissues except for glycogen synthesis in the liver and skeletal muscle in the absorptive phase and gluconeogenesis in the liver and kidney in the fasting phase.

There are four major forms of HK: HK1, HK2, HK3, and HK4 or glucokinase (GCK). The molecular, kinetic, and physiological characteristics of HKs are summarized in **Table 1**. The cellular concentration of the ATP cofactor is typically in the range of 2–8 mM (33). Therefore, HK1, HK2, and GCK function *in situ* with saturating or non-limiting ATP. HK3 of red blood cells (RBCs) operates under ATP-limiting kinetics *in vivo*, which may be moderately enhanced by increased RBC ATP concentration in hyperglycemia (34). The conditions under which HKs function *in situ* with respect to glucose are more complex. From accumulated experimental and clinical evidence discussed below, generally, HK1 is saturated at the levels of intracellular concentration of glucose in all cells in euglycemia and provides a basal level of glucose metabolism (with HK3 providing a similar function in RBCs); HK2 is saturated at the levels of intracellular glucose concentration in euglycemia in some tissues and unsaturated in others with an abnormal, non-transcriptional mechanism circumventing saturation producing dysregulated glycolysis and abnormal accumulation of damaging glycolytic intermediates in persistent hyperglycemia; and GCK, while providing a glucose sensor function for control of insulin and glucagon secretion in pancreatic beta-cells and alpha cells and glycogen synthesis in the liver, also produces abnormal accumulation of damaging glycolytic intermediates and dysregulated glycolysis in persistent hyperglycemia.

**Table 1:** Molecular, kinetic, and physiological characteristics of hexokinases.

Hexokinase isoform	Subunit M_r_ and structure	Kinetics	Principal tissues (% total hexokinase activity)	Comment
Hexokinase-1 (HK1);	100 kDa (α + α_2_)	K_M(Glucose)_ = 61 µM K_M(ATP)_ = 1.2 mM k_cat_ = 100 s^−1^ k_cat_/K_M(Glucose)_ = 1.7 × 10^6^ M^−1^ s^−1^	Adipose (30%), brain (90%), heart (80%–90%), liver (30%), RBCs (50%), skeletal muscle (50%–70%), vascular cells (approximately 50%)	K_i(G6P)_ = 15 µM* Widely expressed. High affinity for mitochondria
Hexokinase-2 (HK2)	100 kDa (α)	K_M(Glucose)_ = 340 µM K_M(ATP)_ = 1.0 mM k_cat_ = 318 s^−1^ k_cat_/K_M(Glucose)_ = 0.9 × 10^6^ M^−1^ s^−1^	Vascular cells (approximately 50%), skeletal muscle (30%–50%), adipose (70%), brain (10%), heart (10%–20%)	K_i(G6P)_ = 210 µM. Insulin-dependent expression. Stabilized to proteolysis at high glucose concentration
Hexokinase-3 (HK3)	100 kDa (α)	K_M(Glucose)_ = 38 µM K_M(ATP)_ = 3.1 mM k_cat_ = 60 s^−1^ k_cat_/K_M(Glucose)_ = 4.2 × 10^6^ M^−1^ s^−1^	RBCs (50%)	K_i(G6P)_ = 130 µM
Hexokinase-4 (HK4) or glucokinase (GCK)	52 kDa (α)	S_0.5 (Glucose)_ = 7,600 µM K_M(ATP)_ = 0.2 mM k_cat_ = 62 s^−1^ k_cat_/K_M(Glucose)_ = 8.2 × 10^6^ M^−1^ s^−1^	Liver (50%)—hepatocytes, pancreatic alpha and beta-cells, intestinal K- and L-cells, hypothalamic neurons, and pituitary cells	Glucose sensor. No G6P inhibition. Functional loss in GCK-MODY. Insulin-dependent expression in the liver

Data from (24–30). *Competitive inhibition of HKs by G6P *in situ* is limited by the much higher intracellular concentration of glucose than G6P and, for HK1, masking of the high-affinity inhibitory binding site to G6P when bound to mitochondria (31)¸ as is normally the case (32).

There is a further minor pathway of glucose metabolism—the polyol pathway. Glucose is metabolized therein by aldose reductase (AR) to sorbitol with onward metabolism by sorbitol dehydrogenase to fructose (35). AR-catalyzed *in situ* kinetics of glucose metabolism are usually <1% flux of total HK activity *in situ*. The polyol pathway has highest activity in the renal medulla (36) where it has a role in osmoregulation, countering extracellular hypertonicity produced during anti-diuresis (37, 38).

## Scheduled and unscheduled glycolysis—importance of increasing activities of glycolytic enzymes to accommodate increased flux of glucose metabolism

**Figure 1:**
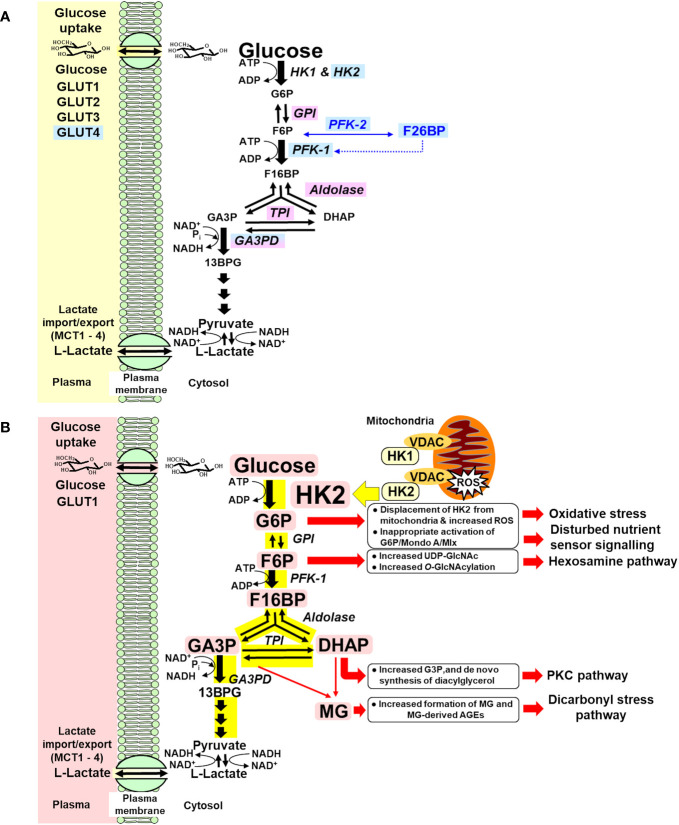
**(A)** Regulation of glycolytic enzymes by insulin and Mondo A in scheduled glycolysis. Blue highlighted text, genes with expression and/or activity regulated by insulin. Lilac highlighted text, genes with expression regulated by Mondo A/Mlx. **(B)** Dysregulation of glycolytic enzymes and metabolic dysfunction in hexokinase-2-linked glycolytic overload and unscheduled glycolysis hypothesis. Pink highlighted text, HK2 and metabolites increased in unscheduled glycolysis. Yellow highlight indicates increased flux in glycolytic overload.

Early-stage glucose metabolism is regulated at three key steps: glucose uptake, hexokinase, and phosphofructokinase (PFK) (39). When increased glucose disposal occurs in the skeletal muscle in the absorptive phase, increased flux of glucose is delivered into the cytoplasm by insulin-stimulated GLUT4 (40) and enters glycolysis by HK1 and HK2 (41), with insulin stimulating increased expression of HK2 (42), producing increased flux of formation and modest increase in the concentration of G6P. G6P is converted to fructose-6-phosphate (F6P) by glucose-6-phosphate isomerase (GPI) and to glucose-1-phosphate for glycogen synthesis by phosphoglucomutase (PGM). Allosteric activation of glycogen synthase by G6P increases its consumption for glycogen synthesis (43). Insulin signaling activates phosphofructokinase-2 (PFK-2), increasing allosteric regulator fructose 2,6-bisphosphate (F26BP) (44), which increases activity of phosphofructokinase-1 (PFK-1) and converts F6P to fructose-1,6-bisphosphate (F16BP). Insulin signaling and G6P-dependent activation of transcriptional signaling of Mlx/Mondo A increases expression and activity of GPI, aldolase, triosephosphate isomerase (TPI), and glyceraldehyde-3-phosphate dehydrogenase (GA3PD) (45–48). The paralog protein, Mondo B or carbohydrate response element binding protein (ChREBP), is dominant in the liver and adipose tissue (49). This provides for regulated increased flux of glucose metabolism from G6P through and beyond glyceraldehyde-3-phosphate (GA3P) with modest increases in the steady-state levels of glycolytic intermediates—scheduled glycolysis (50) (**Figure 1A**). If entry of glucose into glycolysis initiated by HKs occurs without regulatory activation and increased expression of glycolytic enzymes, increased metabolic flux produces concomitant increased steady-state levels of glycolytic intermediates at least proportionate to the increase in flux of glucose metabolism—unscheduled glycolysis. Increase in some glycolytic intermediates—G6P, F6P, dihydroxyacetonephosphate (DHAP), and GA3P—beyond normal tolerable levels stimulates pathways of metabolic and cellular dysfunction (**Figure 1B**). The subversion of the normal gating of glucose entry into metabolism by HKs to produce glycolytic overload and unscheduled glycolysis is the origin of hyperglycemia-induced metabolic dysfunction linked to the development of insulin resistance, beta-cell glucotoxicity, and vascular complications of diabetes.

## Glucose availability and saturation status of hexokinases in cells and tissues in hyperglycemia

Glucose availability for HKs is dependent on the transport of glucose into cells by one or more facilitative glucose transport proteins (GLUTs) and sodium-dependent glucose transporters (SGLTs). The *in situ* activities of glucose transporters and cellular consumption of glucose by HKs usually create a negative concentration gradient of glucose from plasma and interstitial fluid to the cell cytoplasm. This is greatly diminished in cells with a glucose sensor function where intracellular glucose concentrations tracks the extracellular glucose concentration (51) (**Table 2**). The most relevant and important GLUTs for glucose metabolism in impaired metabolic health and glycemic disease are as follows: GLUT1—the major GLUT of vascular cells, non-parenchymal cells of liver, glucose transport of skeletal muscle and adipose tissue in the fasting phase, Schwann cells of peripheral nerve, endothelial and epithelial-like barriers of the brain, fibroblasts, and pancreatic beta-cells; GLUT2—the major GLUT of hepatocytes; GLUT3—the major GLUT of peripheral and CNS neurons; and GLUT4—the major GLUT of skeletal muscle, adipose tissue, and myocardium, with expression also in renal podocytes. In renal tubular epithelial cells, GLUT1 and GLUT2 are expressed along with high expression of sodium-dependent glucose transporter SGLT2, which normally accounts for 90% of reabsorption of glucose passing through the glomerular filter (66). GLUT1 and GLUT3 are high-affinity glucose transporters (K_M(Glucose)_ = 0.7–3.2 mM), GLUT2 is a low-affinity glucose transporter (K_M(Glucose)_ = 17–20 mM), and GLUT4 is a high-affinity glucose transporter (K_M(Glucose)_ = 12.6 mM) with activity dependent on insulin. SGLT2 has high affinity for glucose (K_M(Glucose)_ ≤ 6 mM) and relatively high capacity for glucose transport (66, 89).

**Table 2:** Cytosolic glucose concentration, hexokinase saturation, and increased flux of glucose metabolism in cells and tissues suffering metabolic dysfunction in hyperglycemia.

Cell type	Major GLUT expression	Major HK expression	Ambient glucose	Cytosolic glucose concentration	HK saturation with glucose	ΔFlux (high glucose model)
Cells and tissues at sites of development of vascular complication of diabetes						
Aortal endothelial cells	GLUT1 (52)	HK1 and HK2 (25)	5 mM	8.2 pmol/µg protein (1.0 mM)	HK1, 94%; HK2, 75%	+2-fold (20 mM glucose) (25, 53)
			30 mM	55.2 pmol/µg protein (6.5 mM) (52)	HK1, 99%; HK2, 95%	
Retinal pericytes	GLUT1 (54)	HK1 and HK2 (55)	5 mM	0.2 µg/10^6^ cells (0.6 mM)	HK1, 91%; HK2, 64%	+2-fold (30 mM glucose) (57)
			55 mM	0.5 µg/10^6^ cells (1.5 mM) (56)	HK1, 96%; HK2, 82%	
Mesangial cells	GLUT1 and SGLT2 (58, 59)	HK1 and HK2 (60)	5 mM	0.7 mM	HK1, 92%; HK2, 67%	+3-fold (20 mM glucose) (61)
			20 mM	6.7 mM (61)	HK1, 99%; HK2, 82%	
Renal podocytes	GLUT1 and GLUT4 (62)	HK1 and HK2 (63, 64)	5.6 mM	110 µM/µg protein (0.44 mM)	HK1, 88%; HK2, 56%	Not determined
			35 mM	606 µM/µg protein (2.4 mM) (65)	HK1, 98%; HK2, 88%	
Renal proximal tubular epithelial cells	GLUT1, GLUT2, and SLGT2 (66)	HK1 and HK2 (67)	5.6 mM 25 mM	Not determined	Not determined	+2-fold (68)
Sciatic nerve (healthy control and diabetic rats)/Schwann cells	GLUT1 (Schwann cells) and GLUT3 (Schwann cells and neurons) (69)	HK1 (neurons and Schwann cells) and HK2 (Schwann cells) (70, 71)	5.8 mM	3.4 mM (healthy control rats)	HK1, 98%; HK2, 91%	+ 2-fold in Schwann cells (25 mM glucose) (71)
			18.8 mM (blood)	13.9 mM (diabetic rats) (72)	HK1, 100%; HK2, 98%	
Cells and tissues at sites of development of insulin resistance						
Human leg muscle *in vivo* (^13^C/^31^P NMR)	GLUT1 and GLUT4 (40)	HK1 and HK2 (41)	5 mM	0.25 mM (73)	HK1, 80%; HK2, 42%	+2-fold fasting phase in T2DM (75)
			16.4 mM	0.43 mM (74)	HK1, 88%; HK2, 56%	
3T3 Fibroblast-derived adipocytes	GLUT1 and GLUT4 (76)	HK1 and HK2 (76)	5 mM	*ca.* 0.5 mM	HK1, 89%; HK2, 60%	+2-fold glycolysis
			10 mM	*ca.* 0.7 mM (77)	HK1, 92%; HK2, 67%	
Human hepatocytes in 3D culture Rat liver—glucose loading after fasting Human liver (normal healthy human subjects; hyperglycemic clamp)	GLUT2 (78) GLUT1 (non-parenchymal cells) and GLUT2 (hepatocytes) (78) GLUT1 (non-parenchymal cells) and GLUT2 (hepatocytes) (79)	GCK (70) HK1 and HK2 (non-parenchymal cells) (80) and GCK (hepatocytes) (70) HK1 and HK2 (non-parenchymal cells) and GCK (hepatocytes) (29)	5.5 mM 11 mM	Not determined	Not determined	+3-fold increase (82)
			6.1 mM 14.8 mM (portal vein) (81) 5 mM 7.5 mM 9 mM	9.0 mM 17.2 mM (81) Not determined	GCK, 54% GCK, 69%	+3-fold (glycogen synthesis) (81)
						Increased glucose uptake and oxidation; decreased glucose production and lipid oxidation (83)
Cell model of pancreatic beta-cell glucotoxicity						
MIN6 insulinoma pancreatic beta-like cells	GLUT2 (84)	GCK (84)	5 mM 10 mM	3 mM 10 mM (85)	GCK, 28% GCK, 57%	+2-fold increase (with insulin secretion)

Cell volumes assumed: GM7373 endothelial cells, 1.7 fl (86); retinal pericyte, 1.9 fl per cell (87); and rat mesangial cell, 3.0 fl (88). Percentage saturation of HKs at intracellular glucose concentration is deduced from the Michaelis–Menten equation: saturation (%) = 100 × [Glucose]_Cellular_/(K_M_ + [Glucose]_Cellular_).

Consider initially the glycemic status of HKs at the sites of development of vascular complications of diabetes. In vascular endothelial cells, retinal pericytes, and renal cells, glucose uptake occurs mainly by GLUT1, and hexokinases therein are HK1 and HK2 (**Table 2**). In vascular endothelial cells, under model euglycemic conditions with 5 mM glucose, the estimated intracellular glucose concentration is 1.0 mM producing high saturation of both HK1 and HK2 (94% and 75%, respectively). In model hyperglycemia with high glucose concentration in cell cultures, the intracellular glucose concentration was increased to 6.5 mM with both HK1 and HK2, again highly saturated (99% and 95%, respectively). Despite the high saturation of HKs with glucose, the flux of glucose metabolism in model hyperglycemia increased twofold, compared to the euglycemic control. The increased flux of glucose metabolism occurs without increased expression of HKs, as judged by HK1 and HK2 mRNA and, because of high saturation of HKs, cannot be accounted for by mass action or increased specific turnover at HKs (25). Similar saturation of HK1 and HK2 by intracellular glucose concentration in model euglycemia was found in retinal pericytes and renal mesangial cells, podocytes, and proximal tubular epithelial cells with a two- to threefold increase in flux of glucose metabolism in high glucose concentration. HK saturation by glucose is circumvented by a non-transcriptional increase in HK2 protein, which drives dysfunctional glucose metabolism and pathogenesis in the development of vascular complications of diabetes—see below.

In the peripheral nerve, glucose uptake occurs by GLUT3 in neurons and GLUT1 and GLUT3 in Schwann cells (69). HK1 is expressed in neurons and HK1 and HK2 in Schwann cells and also likely in endoneurial endothelial cells and fibroblasts (70, 71). Studies of the sciatic nerve of healthy control and streptozotocin (STZ)-induced diabetic rats indicate a high glucose content of the nerve, indicating that HK1 and HK2 are saturated in both euglycemia and hyperglycemia. Nevertheless, incubation of Schwann cells in high glucose concentration produced a twofold increase in glucose metabolism (71). This increased flux of glucose metabolism supports metabolic dysfunction in Schwann cells, which likely impairs functional support of axons implicated in the development of peripheral diabetic neuropathy (14).

In peripheral insulin resistance, there is abnormal glucose uptake and metabolism by the major target tissues of insulin, skeletal muscle, and adipose tissue. Glucose uptake by skeletal muscle cells is mediated mainly by GLUT1 in the fasting phase and GLUT4 in the absorptive phase (40). Intracellular glucose concentrations of skeletal muscle of the leg in low and high glucose were 0.25 mM and 0.43 mM, respectively (73, 74). This suggests that HK1 is saturated with glucose, whereas HK2 is not. In patients with T2DM and insulin resistance with FPG increased twofold, there was an approximately twofold increase in glucose metabolism by skeletal muscle (75). This suggests that there is increased non-insulin-mediated glucose uptake (NIMGU) by mass action effect of increased FPG, increasing glucose uptake by GLUT1 and glucose metabolism by HK2 (75, 90). This occurs without increased expression of HK1 or HK2 at the mRNA and protein level (91–94). In the fasting phase, skeletal muscle accounts for 80%–90% of glucose uptake in euglycemia, with most occurring via NIMGU (6, 90). In the absorptive phase, a larger insulin-dependent, GLUT4-mediated glucose uptake and metabolism by skeletal muscle occurs, which is decreased in insulin resistance (75, 90) (**Table 2**).

The glycemic status and glucose metabolism in adipocytes is similar to that of the skeletal muscle. In the adipose tissue, evidence from murine 3T3 fibroblast-derived adipocytes in low and high glucose concentration cultures indicate that HK1 is saturated by glucose in low and high glucose concentration, whereas HK2 is not (77). Under these conditions, there is a twofold increase in glucose metabolism without increase in expression of HK1 and HK2 (76, 95) (**Table 2**).

Hepatic insulin resistance is an important contributor to whole body insulin resistance and increased FPG (96). Glucose uptake by the liver occurs mainly by GLUT2 in hepatocytes and by GLUT1 in non-parenchymal cells (78). In hepatocytes, the metabolism of glucose by GCK accounts for approximately 95% glucose metabolism (97). GCK is not saturated by glucose but is partially inhibited by glucokinase regulatory protein (GKRP) (**Table 2**).

GKRP mRNA levels are high in the liver but low in most other tissues. In the liver, GKRP is expressed at the protein level: a 68-kDa polypeptide that functions as a competitive inhibitor of glucose binding to GCK. At low glucose concentrations, GCK binds to GKRP, and the inactive complex is translocated to the hepatocyte nucleus. When glucose concentration increases, GCK-GKRP complex dissociates and GCK returns to the cytosol to participate in glucose metabolism (98, 99). In human subjects, the formation of the GCK-GKRP complex is promoted by F6P and antagonized by fructose-1-phosphate (F1P), indicating that diets rich in fructose with formation of hepatic F1P can increase GCK activity.

Glucokinase has a low affinity for glucose (S_0_._5 =_ 7.6 mM) and sigmoidal kinetics with respect to glucose, reflecting positive cooperativity with a Hill coefficient of 1.7 (26). Increasing glucose levels in hepatocytes in culture produces modest increases in cellular G6P and marked increases in glycogen synthesis and glycolysis (100). Hepatic glycogen synthase is controlled by interplay between allosteric activation by G6P and reversible phosphorylation through glycogen synthase kinase-3 and protein phosphatase 1 (101). Glycogen synthesis correlates positively with G6P, increasing rapidly at 0.2–0.3 mM G6P where it is like an on–off switch for glycogen synthesis (102), increased G6P synergizing with glucose to induce activating dephosphorylation of glycogen synthase and inactivating dephosphorylation of glycogen phosphorylase (103). There is a consequent sigmoidal relationship between plasma glucose concentration and hepatic glycogen synthesis (104). Hepatic glycolytic flux correlates positively with *in situ* GCK activity (100). High glucose concentration induced repression of GCK and increased expression of G6Pase to decrease G6P and safeguard hepatic phosphate homeostasis (105). The induction of G6Pase is attributed to the activation of ChREBP-Mlx by increased G6P, F26BP, and xylulose 5-phosphate (49, 106). In a hepatocyte 3D primary culture model, the flux of glucose metabolism increased disproportionately (threefold) with twofold increased glucose concentration (82).

Glucose loading by the liver of normal healthy rats showed that increasing portal venous glucose approximately twofold produced a threefold increase in glucose metabolism for hepatic glycogen synthesis (81). Increased glucose uptake by the liver is associated with unchanged or decreased G6P concentration and other glycolytic intermediates, as activities of enzymes of gluconeogenesis are decreased and those of glycogen synthesis and glycolysis are increased (107). The liver responds to increased blood glucose levels in the postprandial state by uptake of glucose and conversion to glycogen (101). The formation of the GCK-GKRP complex and its stabilization by G6P limit the net hepatic glucose uptake and metabolism therein after oral ingestion of glucose (103, 108). By contrast, most fructose is readily taken up from the circulation by the liver, due to the low K_M_ and high *in situ* activity of hepatic ketohexokinase (KHK) (109).

In pancreatic beta-cells, recent evidence suggests that glucose uptake by human beta-cells occurs mainly by GLUT1, whereas in rodent models, it occurs mainly by GLUT2 (110). In human and rodent beta-cells, GCK is the dominant HK (111). Intracellular glucose concentration has been reported for the murine insulinoma MIN6 cell line with glucose uptake by GLUT2 and glucose metabolism mainly by GCK (84, 112). Intracellular glucose concentration is similar to extracellular glucose concentration (85), glucose metabolism is not rate limited by GLUT2 transport (112), and flux of glucose metaboolism increases proportionately to extracellular glucose concentration through physiological range—GCK is not saturated by glucose and is coupled to the secretion of insulin (84). This is consistent with the glucose sensor function of GCK in pancreatic beta-cells (51). In subjects with normal insulin sensitivity and beta-cell function, temporary increases in plasma glucose in the absorptive phase produces increased G6P and other glycolytic intermediates, but these are corrected following insulin secretion and return of plasma glucose concentrations to the basal state in fasting phase. Sustained increased glycolytic intermediates in beta-cells is implicated in glucotoxicity (113). Although little investigated, other cells with GCK expression may be susceptible to glycolytic overload with contribution to glucotoxicity and cell dysfunction—pancreatic alpha-cells, selected neurons of the hypothalamus and brainstem, in the pituitary, and entero-endocrine K and L cells (26, 114, 115).

For other sites of critical glucose metabolism, for example, in neurons of the central nervous system (CNS) and cardiomyocytes of the heart, an abundant pool of intracellular glucose is maintained with glucose metabolism limited by dominant expression of HK1 (27, 116, 117). In the CNS, glucose enters neurons predominantly by GLUT3 (118), and the intracellular concentration of glucose in euglycemia is approximately 1 mM (119). This indicates that HK1 is 94% saturated by intracellular glucose. In cardiomyocytes, glucose uptake is mediated by GLUT1 and GLUT4 (120), intracellular glucose concentration is 1–2 mM (121), and HK1 is 94%–97% saturated by glucose. In these cells, HK1-mediated glucose metabolism prevents glycolytic overload and unscheduled glycolysis when intracellular glucose concentration is increased in hyperglycemia. Impairment of CNS and cardiac function in hyperglycemia may arise through HK2-linked glycolytic overload of vascular cells in blood vessels of the brain and heart, see below, and through the development of cardiomyocyte dysfunction, such as in atrial dilatation, which is associated with increased expression of HK2 (27), increasing the vulnerability to HK2-linked metabolic overload. This may apply particularly in ischemia reperfusion injury with G6P accumulation from glycogenolysis, as reviewed (122).

## Hexokinase-2-linked glycolytic overload and unscheduled glycolysis as the initiator of metabolic dysfunction in the development of vascular complications of diabetes

Increased flux of glucose metabolism in high glucose concentration circumventing glucose saturation of HKs is the key driver of metabolic dysfunction in human endothelial cells, pericytes, renal mesangial cells, podocytes and tubular epithelial cells, Schwann cells of peripheral nerves, and fibroblasts (25, 57, 61, 68, 71, 123). Both HK1 and HK2 expessed in these cell types are saturated with glucose, and yet, the flux of glucose metabolism is increased without increased HK expression in hyperglycemia (25). The explanation for this is stabilization of HK2 protein to degradation by high intracellular glucose concentration (25). HK2 uniquely has a second glucose-binding active site in the C-terminal domain (24). This additional active site contains a degradation motif, _712_QRFEK_716_, that binds heat shock protein cognate 70 (HSC70) and directs HK2 for proteolysis by chaperone-mediated autophagy (124). As plasma glucose concentration increases beyond the normal physiological range, there is a concomitant increase in intracellular glucose concentration. At the C-terminal active site, glucose increasingly competes with HSC70 for binding of the active site and thereby slows the degradation and increases the half-life of HK2. HK2 protein and hexokinase activity consequently increase without change in expression of other glycolytic enzymes downstream in early-stage glycolysis, producing unscheduled glycolysis (122).

Unscheduled glycolysis drives pathways of metabolic dysfunction in cells expressing HK2 in hyperglycemia with high intracellular glucose concentration. The consequences of this are:

(i) Increased levels of G6P, which displace HK2 from mitochondria, impairing disposal of ATP, producing mitochondrial membrane hyperpolarization, mitochondrial dysfunction, and increased formation of reactive oxygen species (ROS) (32, 125); and increased G6P/Mondo A/Mlx transcriptional activity, stimulating lipogenic and other gene expression with regulatory carbohydrate response elements (ChRE) (47);
(ii) Increased F6P and activation of the hexosamine pathway, increasing enzymatic protein glycosylation (126, 127);
(iii) Increased GA3P and DHAP, leading to increased formation and accumulation of the reactive dicarbonyl metabolite, methylglyoxal (MG) or dicarbonyl stress (25, 126, 127), with increased formation of MG-derived advanced glycation endproducts (AGEs) in proteins, which are misfolded proteins and activate the unfolded protein response (UPR) (25, 128);
(iv) Increased glycerol-3-phosphate (from increased DHAP), leading to increased formation of diacylglycerol *de novo* and abnormal activation of protein kinase c (PKC) (126, 127); and
(v) Increased metabolic channeling of G6P for glycogen synthesis as a consequence of displacement of HK2 from mitochondria (32) (**Figure 1B**).

Evidence of all of these effector pathways and biomarkers of HK2-linked glycolytic overload and unscheduled glycolysis has been found in diabetic endothelial dysfunction, DKD, diabetic retinopathy, and diabetic neuropathy (**Table 3**).

**Table 3:** Evidence of hexokinase-2 linked glycolytic overload and unscheduled glycolysis in glycemic disease.

Phenotype	Tissue/cell type	Indications	References
Diabetic endothelial dysfunction	Endothelial cells	Increased glucose metabolism in hyperglycemia through stabilization of HK2 to proteolysis. Glycogen accumulation induced by high glucose concentration *in vitro* and hyperglycemia *in vivo*. Downstream metabolic dysfunction (DS, HP, MD, OS, PKC)*	(25, 126, 127, 129)
Diabetic nephropathy	Renal mesangial, cells, podocytes, and tubular epithelial cells	Increased HK2 protein in human mesangial cells by high glucose concentration *in vitro*. Abnormal glycogen deposition in proximal tubules. Downstream metabolic dysfunction (DS, HP, MD, OS, PKC, Mondo A/Mlx activation)	(130–136)
Diabetic retinopathy	Muller cells, endothelial cells, and pericytes (also intact retina)	HK2 expression in human retina. Abnormal glycogen accumulation. Downstream metabolic dysfunction (DS, HP, MD, OS, PKC)	(55, 135, 137–140)
Diabetic neuropathy	Schwann cells (also dorsal root ganglia and sciatic nerve)	Increased HK2 in hyperglycemia. Glycogen accumulation associated with demyelination and axonal degeneration in clinical diabetic neuropathy. Downstream metabolic dysfunction (DS, MD, OS)	(71, 141–145)
Peripheral insulin resistance	Whole body NIMGU in patients with T2DM and healthy controls	NIMGU—major pathway for glucose disposal in the fasting state; increased approximately twofold in patients with T2DM. Mass action driving increased GLUT1-mediated glucose uptake and HK2-mediated metabolism of glucose in skeletal muscle and adipose tissue	(90)
	Basal and insulin-stimulated whole body and leg glucose disposal in patients with T2DM and healthy controls	Basal (fasting) glucose uptake by the leg tissues was greater in patients with T2DM vs. healthy controls: 1.7 ± 0.2 vs. 1.1 ± 0.1 mg/kg leg wt per min; +55%—GLUT1-mediated NIMGU and HK2-mediated glucose metabolism of leg skeletal muscle	(75)
	Skeletal muscle of transgenic and mutant mice	Overexpression of GLUT1 and overexpression of HK2 (with HFD feeding) produces insulin resistance—increasing HK2-linked unscheduled glycolysis in the fasting state. HK2(+/−) mice had lower plasma glucose and insulin concentrations, compared to WT controls in glucose tolerance test—decreasing HK2-linked unscheduled glycolysis.	(146–148)
	Skeletal muscle in patients with T2DM	Downstream metabolic dysfunction (MD and ROS, HP, DS, Mondo A/Mlx activation and lipogenesis).	(149–152)
	Adipose tissue, insulin-resistant 3T3-L1 adipocytes *in vitro*	HK2 expression, increased glycogen deposition in adipose tissue and downstream metabolic dysfunction (MD and ROS, PKC, DS, Mondo A/Mlx activation and lipogenesis)	(153–158)
Hepatic insulin resistance	Liver of (fa/fa) obese rats	Increased hepatic G6P, F6P, DHAP, and GA3P, indicative of glycolytic overload	(159)
	Human hepatocytes in 3D culture	Incubation of human hepatocytes in high glucose concentration increases hepatic G6P, decreases GCK, and increases GKRP and G6Pase	(105)
	Rat hepatocytes and liver	High glucose concentration increases glycolysis and F26BP/ChREBP/Mlx-linked gene expression, including G6Pase, lipogenic genes, and others	(107) (49)
	Healthy human subjects with normal glucose tolerance	Infusion of glucose to raise plasma glucose by 2.2 mM for 48 h induced hepatic insulin resistance, proposed due hepatic glycolytic overload	(160)
Beta-cell glucotoxicity	Islets of (db/db) diabetic mice, βV59M mutant diabetic mice and normal healthy controls	Increased G6P, F6P, DHAP and GA3P in islets of diabetic mice, compared to non-diabetic controls; and also in islets from non-diabetic mice incubated in high glucose concentration, compared to euglycemic concentration control	(113, 161)
	Rat islets	Overexpression of glutamine:fructose-6-phosphate amidotransferase or treatment with glucosamine results impaired glucose-stimulated insulin secretion and reduced expression of beta-cell specific genes (insulin, GLUT2, and glucokinase).	(162)
	MIN6 cells and pancreas of HFD-fed mice	Increased flux of formation of MG in high glucose concentration cultures of MIN6 cells and increase pancreatic MG in HFD-fed mice	(163)

*Key for effector pathways: DS, dicarbonyl stress; HP, hexosamine pathway; MD, mitochondrial dysfunction; OS, oxidative stress; PKC, protein kinase c pathway.

The initiating event is stabilization of HK2 to proteolysis and unscheduled glycolysis. In confirmation of this, partial silencing or pharmacological treatment to maintain normal levels of HK2 in high glucose concentration prevented high glucose concentration-induced metabolic dysfunction (25, 123). A condition for the induction of glycolytic overload in hyperglycemia is that HK2 is a major contributor to HK activity at the sites of development vascular complications of diabetes. This is indeed found in vascular endothelial cells and pericytes, renal mesangial cells, podocytes and tubular epithelial cells, and Schwann cells of peripheral nerves (55, 60, 63, 64, 67, 71) (**Table 3**).

The extent of increase in HK2 protein by stabilization to proteolysis in hyperglycemia depends on: (i) the increase in cellular glucose concentration such that glucose competes effectively with HSC70 at the C-terminal active of HK2 and (ii) the duration of increased glucose concentration with respect to the *in situ* half-life t_1/2_ of HK2 protein. Our recent studies in endothelial cells indicate that the apparent inhibitor constant K_i_ value of extracellular glucose concentration for inhibition of proteolysis of HK2 is approximately 7 mM (which may vary with differing rates of cellular uptake of glucose and levels of expression of HSC70), and the half-life of HK2 protein is approximately 10 h, indicating that prolonged exposure of HK2 to increased cytosolic glucose concentration in IFG and diabetes is the condition required to stimulate HK2-linked glycolytic overload and unscheduled glycolysis. Independent estimates of the half-life of HK2 are <12 h and 11 h (164, 165). The effect of cellular glucose concentration on HK2 stability to proteolysis was identified previously by others, but the relevance to hyperglycemia and diabetes was not appreciated (124).

An interesting consequence of HK2 displacement from mitochondria in unscheduled glycolysis is increased flux of glucose metabolism for glycogen synthesis by effects of metabolic channeling (32). In human aortic endothelial cells, we found a sevenfold increase in glycogen deposition in high glucose concentration cultures (25), an effect reported previously by others without explanation (166). For *in vivo* translation of these findings, increased glycogen deposition was found in the aorta (129), retina (167, 168), and renal tubular epithelial cells and glomeruli of STZ rats (169). Increased glycogen deposition was also found in Schwann cells and axons of perpiheral nerve in experimental and clinical diabetes (with possible confounding effects of axonia) (141, 170). Indeed, glycogen deposition in the kidney of patients with diabetes has long been known and called “Armanni–Ebstein” diabetic nephropathy, linked to severity of disease (171). Increased glycogen deposition at the sites of vascular complications of diabetes is explained by the HK2-linked glycolytic overload and unscheduled glycolysis hypothesis.

Recent studies suggest that increased activation of Mondo A/Mlx in hyperglycemia contributes to endothelial dysfunction in diabetes and diabetic kidney disease. In vascular endothelial cells, high glucose concentration stimulated G6P/Mondo A/Mlx-induced increased expression of thioredoxin interacting protein (TXNIP) (172). Increased TXNIP synergizes with activation of the UPR, also activated in hyperglycemia (25), to stimulate inflammatory signaling via the NLRP3 inflammasome (173). Knockdown of Mondo A in podocytes decreased albuminuria, mesangial expansion, and glomerular basement membrane thickening in mutant leptin receptor (db/db) insulin-resistant, diabetic mice (174). Mondo A had increased *O*-GlcNAc enzymatic glycosylation through hexosamine pathway activation with increased stability to proteolysis, nuclear translocation, and transcriptional activity in mesangial cells incubated in high glucose concentration and mesangial of streptozotocin-induced diabetic rats. This was linked to increased lipid deposition and glomerulosclerosis (175). By these mechanisms, the activation of Mondo A during glycolytic overload may contribute to the damaging effects of hyperglycemia at vascular sites.

## Hexokinase-2-linked unscheduled glycolysis as initiator of peripheral insulin resistance

The skeletal muscle is the major site of insulin-mediated glucose uptake in the postprandial state and the major tissue involved in direct catabolism of glucose (176, 177). Hence, the dysfunction of glucose uptake and metabolism by the skeletal muscle has a major impact on glucose homeostasis (178).

The skeletal muscle and adipose tissue have relatively high expression of HK2 (41, 76). Glucose entry into myocytes and adipocytes is mediated by GLUT1 in the fasting phase and insulin-dependent GLUT4 in the absorptive phase. A primary characteristic of insulin resistance is decreased recruitment of GLUT4 to the cell surface in response to insulin due to impaired GLUT4 trafficking, as recently reviewed (179). GLUT1 mRNA and protein in the skeletal muscle was unchanged, increased, or decreased in insulin resistance (40, 180, 181); when decreased, the decrease was more than compensated by the mass action effects of increased FPG in the *in situ* rate of glucose uptake (181). Unlike vascular cells, the intracellular concentration of glucose in myocytes and adipocytes is usually relatively low: HK1 is saturated by glucose euglycemia and hyperglycemia, whereas HK2 is not. Insulin increases the expression of HK2 in the skeletal muscle (42) and also binding of HK2 to mitochondria, likely by increasing phosphorylation by protein kinase B, also called Akt (182, 183). There is no indication of increased HK2 expression at the mRNA and protein level in the skeletal muscle and adipose tissues in clinical insulin resistance and some reports of decreases (91–94, 184, 185), likely due to decreased insulin-dependent expression (91). In the fasting phase, patients with insulin resistance and T2DM had higher glucose metabolism in the skeletal muscle, compared to healthy controls, mediated by mass action increased NIMGU (75, 90). As this occurs without increased insulin and other regulatory signaling, the conditions for HK2-linked unscheduled glycolysis are established. Clinical studies and experimental functional genomics studies of HK2 and GLUT1 in the skeletal muscle of mice support this, as previously discussed (186) and summarized herein in **Table 3**.

Overexpression of HK2 in the skeletal muscle induced insulin resistance in mice fed a high fat diet (HFD) (146). This finding was contrary to the hypotheses tested that “impaired muscle glucose uptake resulting from high fat feeding would be exposed in high flux states and could be corrected by HK2 overexpression.” In addition, heterozygous knockdown of HK2 in mice improved hyperglycemia and hyperinsulinemia (147), as we previously described (186). These findings are consistent with an inverse relationship between insulin sensitivity and HK2 expression, predicted from the hypothesis of HK2-linked unscheduled glycolysis contributing to the development of insulin resistance.

The overexpression of GLUT1 in the skeletal muscle of mice induced insulin resistance (148). We interpret this as increasing glucose uptake in the skeletal muscle in euglycemia—particularly in the fasting phase—and thereby mimicking the effect of mass action increase in glucose uptake by the skeletal muscle in increased FPG found in clinical insulin resistance (75). In the GLUT1 overexpressing mouse, there was increased intracellular glucose concentration, increased HK activity, and increased deposition of glycogen in the skeletal muscle—the latter not due to increased glycogen synthase or decreased glycogen phosphorylase activities (187). Impaired recruitment of the GLUT4 in response to insulin was linked to increased *O*-GlcNAcylation of membrane proteins, including GLUT4 (188). These effects are explained by HK2-linked unscheduled glycolysis.

In contrast, overexpression of GLUT4 in the skeletal muscle of mice did not induce insulin resistance (189). We interpret this as increasing insulin-stimulated GLUT4-mediated glucose uptake, occurring together with insulin- and G6P/Mondo A/Mlx-linked increase in the activity of HK2 and other glycolytic enzymes. This thereby does not produce unscheduled glycolysis and thereby does not induce insulin resistance. The transgenic mouse model with overexpression of both GLUT1 and HK2 together in the skeletal muscle was characterized by a threefold increased G6P concentration and 7.5-fold increased glycogen levels in the skeletal muscle on a standard diet, with no change in basal or insulin-stimulated whole-body glucose disposal (190). We interpret this model as establishing conditions for chronic exposure of skeletal muscle to increased G6P, inducing increased transcription of glycolytic enzymes through G6P/Mondo A/Mlx and thereby negating unscheduled glycolysis. HK2 overexpression gave markedly increased cytosolic HK2, producing increased glycogen by metabolic channeling. This may indicate that clinical HK2-linked unscheduled glycolysis is exacerbated by variability in hyperglycemia, with glycolytic intermediates accumulating most markedly with increased plasma glucose concentration after a period of lower glucose exposure and low level G6P/Mondo A/Mlx transcriptional signaling with low basal expression of glycolytic enzymes.

Evidence of activation of effector pathways of HK2-linked unscheduled glycolysis in peripheral insulin resistance has been reported: in the skeletal muscle, mitochondrial dysfunction, increased ROS formation, hexosamine pathway, MG-derived AGEs, and increased Mondo A/Mlx signaling have been found in insulin resistance (149–152) and in adipose tissue, mitochondrial dysfunction, increased ROS formation, increased PKC activity, dicarbonyl stress, and increased Mondo A/Mlx signaling (153–156) (**Table 3**).

## Glucokinase-linked glycolytic overload and unscheduled glycolysis in hepatic insulin resistance and beta-cell glucotoxicity

GCK is the major hexokinase in hepatocytes and beta-cells, and both have efficient glucose uptake by GLUT2 and GLUT1 glucose transporters, respectively, with increased plasma glucose producing similar increases in intracellular glucose (**Table 2**). This reflects the role of GCK in the liver and beta-cells as glucose sensors to store or release glucose and secrete insulin in response to high glucose concentration, respectively (51). These cells are susceptible to glycolytic overload and unscheduled glycolysis because the low affinity of GCK for glucose produces increased flux of glucose metabolism with increasing intracellular glucose concentration. There is also uptake of dietary fructose by GLUT5 and metabolism by KHK to F1P in hepatocytes (191), which supplements the triosephosphate pool after cleavage to DHAP and glyceraldehyde by aldolase, with the latter phosphorylated to GA3P by triokinase, and increased formation of MG (192).

Hepatic insulin resistance is defined as raised basal hepatic glucose production in the presence of normal or raised plasma insulin levels, suggesting a failure of normal suppression of hepatic glucose production (193). In experimental obese, hyperglycemia leptin receptor mutant (fa/fa) rats with hepatic insulin resistance, there are increases in hepatic G6P, F6P, DHAP, and GA3P, indicative of glycolytic overload (159). Studies of human hepatocytes incubated in high glucose concentration *in vitro* suggest that glycolytic overload is countered by decreased expression of GCK and increased expression of GKRP and G6Pase, all serving to decrease the concentration of G6P (105). Increased glucose metabolism was influenced 45% by extracellular glucose concentration and 55% by insulin, the latter decreasing in an experimental model of insulin resistance (82). In normal healthy rats, increase in plasma glucose produces increased hepatic glycogen synthesis and glycolysis and decreased glucose production. Hepatic G6P was normal or lower than normal and the glycolytic regulator F26BP was increased (107). In hepatocytes, F26BP is a cofactor for ChREBP/Mlx for the induction of expression of G6Pase, lipogenic, and other ChRE target genes (49), indicating that persistent increased glucose metabolism through glycolysis in the liver stimulates increased hepatic glucose production and *de novo* lipogenesis. Indeed, increased hepatic *de novo* lipogenesis is a feature of clinical insulin resistance (194). Increased hepatic DHAP and GA3P (159) leads to increased formation of MG, accounting for the observed MG-derived AGE, hydroimidazolone MG-H1, in HFD-fed rats (195) and stimulus for the activation of the UPR (25) [which also increases hepatic G6Pase (196)]. Prolonged incubation of human hepatocytes with fructose and fatty acids decreased glucose consumption and induced G6Pase, ChREBP, and fatty acid synthase (FASN) (82). Infusion of glucose in healthy human subjects with normal glucose tolerance to raise plasma glucose by 2.2 mM for 48 h induced hepatic insulin resistance (160). We hypothesize that persistent increase in plasma glucose overwhelms the control of hepatic G6P producing hepatic glycolytic overload, simulating expression of G6Pase with increased hepatic glucose production and increased lipogenesis through activation of ChREBP/Mlx.

Activation of ChREBP by increased hepatic G6P, however, limits the increase in hepatic phosphorylated glycolytic intermediates and maintains levels of ATP during hyperglycemia (105). It may thereby be considered protective against glycolytic overload—as previously proposed (197, 198). Indeed, hepatic overexpression of ChREBP prevented the development of insulin resistance and glucose intolerance in HFD-fed mice, although hepatic steatosis increased. Overexpression of ChREBP hepatocytes *in vitro* induced expression of ChRE-regulated target gene, stearoyl-CoA desaturase 1 (Scd1), catalyzing the conversion of saturated fatty acids (SFAs) into monounsaturated fatty acids. SFA impairment of insulin-responsive Akt phosphorylation was thereby rescued through increased Scd1. The expression of G6Pase was also increased (199).

Studies of ChREBP silencing in hepatocytes showed decreased glucose-mediated induction of glycolytic and lipogenic genes and decreased lipid accumulation (200). Global ChREBP-deficient mice had decreased glycolytic and lipogenic gene expression and triglyceride synthesis and increased accumulation of glycogen in the liver under high starch feeding (201). Liver-specific inhibition of ChREBP by shRNA silencing in obese and insulin-resistant, leptin mutant ob/ob mice decreased hepatic steatosis, plasma triglycerides, and non-esterified fatty acids. This improved insulin signaling in the liver, skeletal muscle, and white adipose tissue, with overall improved glucose tolerance and insulin sensitivity (202). Similar effects were found in global homozygous ChREBP knockout ob/ob mice (203).

The protective effects of hepatic ChREBP are most marked in high fructose and sucrose feeding. Hepatic-specific knockout of ChREBP in mice were tolerant to high fructose diets but had increased plasma alanine aminotransferase and aspartate transaminase activities, enlarged liver, and hepatic glycogen overload. Liver G6P was increased approximately twofold, and ATP decreased approximately 40%. Hepatoxicity was rescued by overexpression of liver pyruvate kinase (204). Increased formation of F1P with activation of GCK and loss of ChREBP-induced G6Pase may synergize to increase G6P and produce damaging glycolytic overload.

In clinical studies, ChREBP expression correlates positively with glucose intolerance, insulin resistance, and steatosis, as reviewed (205). This suggests that while a protective role against hepatic glycolytic overload of ChREBP is maintained, the deleterious effects of ChREBP-mediated lipogenesis may be dominant overall during the development of insulin resistance. In the current hypothesis, we also propose a contribution to the development of insulin resistance from the activation of Mondo A in skeletal muscle in hyperglycemia.

Glycolytic overload in pancreatic beta-cells contributes to glucotoxicity—the term referring to the phenomenon of impaired β-cell function during states of elevated glucose concentration (206). In mutant leptin receptor (db/db) insulin-resistant diabetic mice, there was increased G6P, F6P, DHAP, and GA3P in islets, compared to non-diabetic controls (161). Similar effects were found in islets of βV59M mutant inactive beta-cell potassium channel diabetic mice, compared to wild-type controls, and control islets incubated in high glucose concentration (113). This suggests that glycolytic overload and unscheduled glycolysis are established in persistent hyperglycemia of diabetes and high glucose concentration *in vitro*. For downstream effector pathways, beta-cells are resistant to mitochondrial hyperpolarization and increased ROS formation. GCK is attached to mitochondria in a multiprotein complex with high affinity (207), despite high cellular G6P concentration (>1 mM, as judged by INS-1 insulinoma cell line) (208), and mitochondrial protein thiols of beta-cells were reduced rather than oxidized in rat islets in high glucose concentration (209). The overexpression of glutamine:fructose-6-phosphate amidotransferase or treatment with glucosamine to increase hexosamine pathway activity impaired glucose-stimulated insulin secretion and reduced the expression of beta-cell-specific genes (insulin, GLUT2, and glucokinase) (162). Recent studies have indicated that one or more glycolytic metabolites downstream of phosphofructokinase and upstream of GA3PD mediate the effects of chronic hyperglycemia (113). This is consistent with increased DHAP, GA3P, and formation of MG, which may activate the UPR by MG-modified misfolded proteins (25, 128). Indeed, the formation of MG was increased in MIN6 cells incubated in high glucose concentration, and pancreatic MG concentration was increased in HFD-fed insulin-resistant mice (163). Finally, increased Mondo A/Mlx signaling in beta-cells in persistent hyperglycemia is linked to increase in TXNIP (210), inflammation, impaired insulin secretion, and apoptosis (211) (**Table 3**).

In pancreatic beta-cells, there appears to be limited protection from glycolytic overload and unscheduled glycolysis. The expression of GKRP is very low (212) with no evidence for GKRP expression at the protein level, concurring with the lack of accumulation of GKRP in the nucleus of these cells (213). Protection may be afforded by downregulation of expression of GCK and increased expression of other hexokinases, thereby limiting the glycolytic response of beta-cells at high glucose concentration. Prolonged exposure to increased glucose concentration produced by 90% pancreatectomy in rats induced change in HK expression in pancreatic islets—50% decreased expression of GCK and increased expression of other HKs normally considered “forbidden” gene expression in beta-cells: HK1, +4.5-fold; HK2, +20-fold; and HK3, +47-fold (214). Similar trends were found in pancreatic islets of senescence-accelerated mice (SAM) and db/db obese mice: decreased GCK expression and increased HK1–HK3, particularly HK2; 43% and 64% decrease in GCK; and two- and sixfold increased HK2 in SAM and db/db mice, respectively (161). The downside is loss of glucose sensor function, impairing increased insulin secretion in response to increasing glucose concentration, decreasing beta-cell functionality, and progression to development of T2DM. There is also increased vulnerability to HK2-linked glycolytic overload in hyperglycemia. Mechanisms proposed for this change in islet identity are loss of transcription factor FoxO1 (215), maladaptive UPR (216, 217), and inflammation (218), which may be mediated by the UPR (211) and oxidative stress (219). In clinical translation, increasing FPG within the normal range in subjects with normal glucose tolerance or IGT correlates with decline in hyperglycemia-induced insulin secretion (220).

## Hexokinase-linked glycolytic overload and unscheduled glycolysis versus mitochondrial superoxide overproduction hypotheses of metabolic dysfunction in hyperglycemia

The “hyperglycemia-induced mitochondrial superoxide overproduction” hypothesis has been proposed in explanation of hyperglycemia-induced metabolic dysfunction in the development of vascular complications of diabetes (221), with extension of this applied to insulin resistance and glucotoxicity (222). In this hypothesis, it was envisaged that “hyperglycemia-induced overproduction of superoxide by the mitochondrial electron-transport chain partially inhibits GA3PD, thereby diverting upstream metabolites from glycolysis into pathways of glucose overutilization. This results in increased flux of DHAP to DAG, an activator of PKC, and of triosephosphates to MG. Increased flux of F6P to UDP-N-acetylglucosamine increases modification of proteins by O-linked N-acetylglucosamine and increased glucose flux through the polyol pathway consumes NADPH and depletes GSH.”

A consequence of the mitochondrial superoxide overproduction hypothesis is that the proportion of the flux of glucose metabolism leading to the formation of MG is increased in high glucose concentration. However, in studies of high glucose concentration-induced metabolic dysfunction in endothelial cells and fibroblasts, the proportion of glucose metabolism accounted for as the formation of MG was unchanged (25, 123). This indicates that the *in situ* activity of GA3PD is not inhibited. Increased flux of formation of MG was rather due to increased flux of glucose metabolism through glycolysis by circumvention of HK saturation by glucose-induced stabilization of HK2 to proteolysis (25, 123). Increased flux of glucose metabolism and its gating by HKs was not considered in the mitochondrial superoxide overproduction hypothesis. It is the stabilization of HK2 to proteolysis and unscheduled glycolysis rather than mitochondrial superoxide overproduction that initiates metabolic dysfunction in hyperglycemia. There is also a minor increase in flux of glucose metabolism through the polyol pathway due to the low affinity of AR for glucose, but this only accounts for 1%–2% total glucose metabolism at the site of vascular complications of diabetes, insulin resistance, and beta-cell glucotoxicity.

The mitochondrial superoxide overproduction hypothesis sought to explain mitochondrial dysfunction and related increased formation of ROS as part of hyperglycemia-induced metabolic dysfunction (126), but other key aspects were not considered. The key insights and advances in the new hypothesis are as follows: (i) mitochondrial superoxide overproduction is not the initiator of metabolic dysfunction but rather one of multiple downstream effector pathways, and (ii) contribution of the displacement of HK2 from mitochondria by high cellular G6P as a cause of mitochondrial membrane hyperpolarization is recognized (25, 32, 125). Increased formation of ROS in vascular cells in hyperglycemia also occurs by activation of vascular NADPH oxidase and uncoupling of endothelial nitric oxide synthase, stimulated by activation of PKC—a further effector pathway of glycolytic overload and unscheduled glycolysis (223, 224).

The prevention of metabolic dysfunction induced by high glucose concentration in endothelial cells by the overexpression of uncoupling protein-1 (UCP1) and superoxide dismutase-2 (SOD2) appeared to provide strong support for the hyperglycemia-induced mitochondrial superoxide overproduction hypothesis (126). However, both overexpression of UCP1 and SOD2 have recently been found to decrease cellular ATP (225, 226), and this may have prevented metabolic dysfunction by decreasing *in situ* activity of hexokinase rather than by countering mitochondrial dysfunction and increased ROS formation.

The mitochondrial superoxide overproduction hypothesis held promise of antioxidant therapy in prediabetes and diabetes, which has been unfulfilled. Daily administration of 400 IU vitamin E for 4.5 years to middle-aged and elderly people with diabetes and CVD and diabetic nephropathy and had no effect (227). α-Lipoic acid administered for 2 years produced no benefit against endpoints of diabetic macular edema or best-corrected visual acuity in diabetic retinopathy (228). A meta-analysis concluded that there is weak evidence of benefit of α-lipoic acid treatment in diabetic neuropathy (229). Vitamin E improved insulin resistance in non-diabetic overweight subjects, but the effect appeared transient (230). A meta-analysis of studies of vitamin E and vitamin C, individually or in combination, concluded that there was no evidence for improvement of insulin resistance in diabetes by the treatments (231). Ineffectiveness of antioxidant therapy is envisaged in the HK-linked glycolytic overload and unscheduled glycolysis hypothesis as increased formation of ROS is only one of multiple effector pathways downstream of initiation by HK-gated increase in glucose metabolism.

## Predictions of the hexokinase-linked glycolytic overload and unscheduled glycolysis hypothesis

From the new hypothesis, we expect to provide explanations of previously unexplainable observations and provide new strategies for improved disease management and treatment of glycemic disease. These are summarized in **Table 4**. Explanations may now be provided for the following: (i) susceptibility of peripheral neurons and resistance of CNS neurons to metabolic dysfunction and pathogenesis in hyperglycemia; (ii) progression of DKD and diabetic retinopathy by increased expression of HK2; (iii) increased deposition of glycogen at sites of vascular complications of diabetes linked to the progression of vascular complications; (iv) counterintuitive link of increased HK2 expression in the skeletal muscle to the development of insulin resistance and heterozygous HK2 knockdown to improved insulin sensitivity in mice; (v) FPG being a risk predictor for the progression of insulin resistance; and (vi) patients with T1DM who may also exhibit insulin resistance (**Table 4**).

**Table 4:** Predictions of the hexokinase-linked glycolytic overload and unscheduled glycolysis hypothesis.

Prediction	Evidence	Reference	
Expression of HK2 is risk predictor of DKD progression	High expression of HK2 in dermal fibroblasts was linked to faster progression of DKD in patients with T1DM	(232)	
Retinal expression of HK2 is risk factor for diabetic retinopathy	Mice with diabetic retinopathy had higher expression of HK2. Pathogenesis was decreased by silencing of HK2.	(233)	
Increased deposition of glycogen at sites of vascular complications of diabetes	Increased glycogen deposition in the aorta, tubular epithelial cells and glomeruli, and retina peripheral nerve of STZ diabetic rats. Increased glycogen deposition in the kidney and retina of clinical diabetic nephropathy and neuropathy	(129, 141, 167–171)
Peripheral nerves are sensitive and CNS neurons resistance to the damaging effects of hyperglycemia	Increased FL content of cytosolic protein extracts of brain and sciatic nerve of STZ diabetic rats but increase in MG-derived AGEs only in the sciatic nerve. HK1 expression of neurons prevents glycolytic overload and unscheduled glycolysis, while Schwann cells supporting peripheral neurons are vulnerable.	(234)
HK2 expression is a risk factor for development of insulin resistance	Overexpression of HK2 in skeletal muscle induced insulin resistance in HFD-fed mice and heterozygous knockdown of HK2 in mice improved hyperglycemia and hyperinsulinemia	(146, 147)
Increased FPG is a driver of insulin resistance	Increased FPG was a risk predictor of development of insulin resistance and T2DM, independent of change in 2-h plasma glucose in an oral glucose tolerance test.	(16)
Insulin resistance is a characteristic of subjects with T1DM	Insulin resistance was a prominent feature of patients with T1DM, linked mainly to insulin resistance at peripheral tissues, particularly skeletal muscle.	(178, 235)
Pancreatic alpha-cells, selected neurons of the hypothalamus and brainstem, in the pituitary and entero-endocrine K and L cells suffer glucotoxicity in persistent hyperglycemia	Little investigated. Glucotoxicity induces abnormal glucagon secretion in InR1G cells.	(236)

Predictions to be explored in the future are (i) the positive association of HK2 expression with clinical insulin resistance and (ii) glycolytic overload in cells other than hepatocytes and beta-cells with dominant GCK expression and increased intracellular glucose concentration in hyperglycemia.

For therapeutics development, correcting the primary initiating step of dysfunctional glucose metabolism in hyperglycemia is likely the most effective pharmacological target, that is, dysregulation of HK2 expression. In vascular complications of diabetes, the pharmacological intervention required is to normalize HK2 protein levels to those of euglycemic control. To counter peripheral insulin resistance, we seek to inhibit HK2-mediated glucose metabolism in the skeletal muscle and adipose tissue in the fasting phase but not in the absorptive phase; therefore, direct chemical inhibitors of HK2 are undesirable. We rather need to correct the dysfunctional regulation of HK2 expression. Correcting peripheral resistance is expected to increase glucose disposal, decreasing plasma glucose concentration and thereby indirectly relieving glycolytic overload linked to hepatic insulin resistance and beta-cell glucotoxicity.

An example of a therapeutic agent that corrects HK2-linked metabolic dysfunction in hyperglycemia is the glyoxalase 1 (Glo1) inducer, optimized to a combination of *trans*-resveratrol (tRES) and hesperetin (HESP), or tRES-HESP (237). The combination synergizes at the receptor, transcription factor nuclear factor erythroid 2-related factor 2 (Nrf2), and HESP improves bioavailability of tRES by inhibition of intestinal glucuronosyl-transferases (238). Glo1 catalyzes the detoxification of MG and is the optimum strategy to counter dicarbonyl stress (239). The GLO1 gene has a functional antioxidant response element (ARE) where Nrf2 binds to induce increased expression of Glo1 (240). We developed a therapeutic strategy to optimize compounds for the induction of expression of Glo1 by the activation of Nrf2 with a functional GLO1-ARE reporter assay (237). A further ARE-linked, Nrf2-regulated gene is glucose-6-phosphate dehydrogenase (G6PD). Increasing expression of G6PD decreases the steady-state concentration of G6P, decreasing transcriptional activity of G6P/Mondo A/Mlx and thereby decreasing expression of HK2. The decrease in G6P also decreases displacement of HK2 from mitochondria and related mitochondrial dysfunction. There is emerging evidence that tRES-HESP corrects HK2-linked glycolytic overload and potently prevents vascular cell dysfunction in high glucose concentration.

Incubation of human aortal endothelial cells with tRES-HESP in high glucose concentration decreased HK2 protein levels and corrected the flux of glucose metabolism to those of low glucose concentration control cultures and all metabolic dysfunction (25). Similar responses have been found in other cell types (123). tRES-HESP also prevented bone-marrow-derived progenitor cell dysfunction and improved wound healing in experimental diabetes (241).

tRES-HESP was evaluated clinically in non-diabetic overweight and obese subjects with insulin resistance in a double-blind, placebo-controlled crossover study—Healthy Aging Through Functional Food (HATFF) study. tRES-HESP (90 mg tRES, 120 mg HESP), once daily given by oral capsule, for 8 weeks improved insulin sensitivity to that of lean, healthy controls, improving FPG, vascular inflammation, and dicarbonyl stress (237). Insulin resistance was measured by the oral glucose insulin sensitivity (OGIS) index, which correlates well with the reference hyperinsulinemic–euglycemic clamp technique (242). The magnitude of change in OGIS with tRES-HESP treatment, 42–58 ml min^−1^ m^−2^, was comparable to that achieved with pharmaceutical treatment of patients with T2DM (for example, 1.7 g metformin per day, ΔOGIS = +54 ml min^−1^ m^−2^) (243) and extreme weight loss with gastric band surgery in morbid obesity (ΔOGIS = +62 ml min^−1^ m^−2^) (244). There were also links to two established mediators of insulin resistance: TXNIP and tumor necrosis factor-α (TNFα). TXNIP is a mediator of insulin resistance in the liver, skeletal muscle, and adipose tissue and impaired pancreatic beta-cell insulin secretion (245–247). It has expression induced by G6P/Mondo A/Mlx (248) and therefore has expression-induced downstream of glycolytic overload. It also has expression increased by the activation of the UPR sensor IRE1α, increasing expression of XBP1 and cleavage of miR-17, the latter stabilizing TXNIP mRNA (249). TNFα decreases insulin receptor signaling in the adipose tissue and skeletal muscle, particularly prior to development of T2DM (250–252). tRES-HESP treatment produced a decrease in PBMC TNFα expression in the obese subject subgroup of the HATFF study (237). TNFα expression is likely increased through the activation of the UPR in glycolytic overload with TXNIP linked to the activation of the NLRP3 inflammasome (249). Reversal of insulin resistance by tRES-HESP was not achieved by tRES and HESP individually in clinical evaluation (253, 254), suggesting pharmacological synergism of tRES-HESP.

The correction of insulin resistance by tRES-HESP treatment in the HATFF study is consistent with both improvement of insulin resistance and glucotoxicity (237). The overweight and obese subjects treated likely have reversible impairment of beta-cell function (10). Treatment with tRES-HESP corrected peripheral insulin resistance, relieved dysglycemic stress, and thereby provided conditions for normal beta-cell function to be restored.

Targeting activation of Nrf2 to correct insulin resistance is supported by studies of tissue-specific genetic activation of Nrf2 in HFD-fed mice by partial knockdown of Nrf2 antagonist, Keap1. The activation of Nrf2 in the skeletal muscle prevented insulin resistance, and the activation of Nrf2 in the liver prevented hyperglycemia (255). A similar activation of Nrf2 in all tissues prevented the onset of diabetes in db/db mice (256). Research on the development of activators of Nrf2 for the treatment of vascular complications of diabetes has been advanced (257). In future studies, the development of Nrf2 activators to correct HK2-linked glycolytic overload and unscheduled glycolysis treatments may prove effective where optimizations for the induction of G6PD and Glo1 expression are key ARE-linked gene targets. Countering dicarbonyl stress is linked to the prevention of vascular complications of diabetes and treatment of insulin resistance, as reviewed (239). Increasing G6PD expression is expected to improve beta-cell function and glucose tolerance (258) and decrease progression of vascular complications of diabetes (259–261). Previous considerations have linked G6PD activity to countering oxidative stress but suppression of cellular G6P, Mondo A/ChREBP activation, HK2 expression, and glycolytic overload are likely also involved. Nrf2 activators have differing abilities to induce ARE-linked gene expression by mechanisms not yet fully understood (262), so functional screens of activators for the induction of target gene expression are currently required.

## Concluding remarks

The hexokinase-linked glycolytic overload and unscheduled glycolysis hypothesis recognizes the crucial role of HKs in the initiation of dysfunctional metabolism and pathogenesis in hyperglycemia. When there is persistent increased flux of glucose entering glycolysis without concomitant increase in glycolytic enzyme activities to catalyze its onward metabolism, steady-state levels of glycolytic intermediates exceed tolerable limits and metabolic dysfunction leading to a decline in insulin sensitivity and beta-cell function, and glycemic disease may ensue.

## Data availability statement

The original contributions presented in the study are included in the article/supplementary material. Further inquiries can be directed to the corresponding author.

## Author contributions

NR: Writing – review & editing. PT: Conceptualization, Data curation, Formal Analysis, Investigation, Methodology, Writing – original draft.
